# Drug repurposing based on the DTD-GNN graph neural network: revealing the relationships among drugs, targets and diseases

**DOI:** 10.1186/s12864-024-10499-5

**Published:** 2024-06-11

**Authors:** Wenjun Li, Wanjun Ma, Mengyun Yang, Xiwei Tang

**Affiliations:** 1 Hunan Provincial Key Laboratory of Intelligent Processing of Big Data on Transportation, Changsha University of Science and Technology, Changsha, Hunan China; 2 School of Intelligent Manufacturing, Hunan First Normal University, Changsha, 410205 Hunan China

**Keywords:** Drug repurposing, Drug-Target-Disease ternary relationship, DTD-GNN model

## Abstract

**Motivation:**

The rational modelling of the relationship among drugs, targets and diseases is crucial for drug retargeting. While significant progress has been made in studying binary relationships, further research is needed to deepen our understanding of ternary relationships. The application of graph neural networks in drug retargeting is increasing, but further research is needed to determine the appropriate modelling method for ternary relationships and how to capture their complex multi-feature structure.

**Results:**

The aim of this study was to construct relationships among drug, targets and diseases. To represent the complex relationships among these entities, we used a heterogeneous graph structure. Additionally, we propose a DTD-GNN model that combines graph convolutional networks and graph attention networks to learn feature representations and association information, facilitating a more thorough exploration of the relationships. The experimental results demonstrate that the DTD-GNN model outperforms other graph neural network models in terms of AUC, Precision, and F1-score. The study has important implications for gaining a comprehensive understanding of the relationships between drugs and diseases, as well as for further research and application in exploring the mechanisms of drug-disease interactions. The study reveals these relationships, providing possibilities for innovative therapeutic strategies in medicine.

## Introduction

In practical scenarios, many drugs exhibit diverse effects and mechanisms of action, impacting multiple biological processes. This suggests that a drug’s therapeutic potential may extend beyond its known applications, potentially offering benefits in treating other previously unexplored diseases. This concept underlies the practice of drug repurposing, also referred to as drug reuse. Drug repositioning involves the repurposing of an approved drug, originally intended to treat a specific disease, to treat another disease. This process typically refers to drugs that have already undergone clinical trials and received regulatory approval for their safety and efficacy [1].

In drug retargeting research, the relationship among drugs, targets, and diseases is essential. Binary and ternary relationship models are used to explore these connections. Binary relationships examine pairwise or self-relationships among drugs, targets, and diseases, while ternary relationships involve all three entities.

Numerous studies focus on drug repositioning through binary relationships. Wang et al. [2] introduce CGINet networks, which integrate chemical-gene interactions to reveal drug effects on specific genes. The analysis of protein-protein interaction (PPI) networks is crucial for uncovering drug-disease associations and offers a promising therapeutic strategy by modulating PPIs [3]. Zeng et al. [4] develop a graph neural network model for predicting drug-target associations by integrating multiple features. Cao et al. [5] classify drug-target pairs based on binding affinity using a chemical genomics framework and random forest. Li et al. [6] propose a comprehensive disease-gene association model based on parallel graph transformation networks. These studies contribute to drug repositioning research by exploring various approaches and methodologies. Zhao et al. [7] introduce a novel modality-aware MDA prediction model, MotifMDA, which is capable of achieving highly accurate MDA prediction through the fusion of high-order and low-order structural information. The integration of base-order level structural information enables MotifMDA to identify new MDAs from diverse perspectives.

While these studies have contributed to the field of drug repositioning, they have limitations. They mainly focus on the relationship between drugs and a single target or disease, but ignore the complex ternary relationship among drugs, targets, and diseases.

In addition to the traditional drug-disease and drug-target binary relationships, recent studies have shown the importance of triple drug-target-disease interactions in the human metabolic system [8–10]. Some methods have been proposed to construct ternary relationships, such as tensor factorisation to infer missing drug-target-disease tensor entries [8, 10, 11], Qu [12] et al. develop the concept of event graphs and use nodal prediction methods to study drug repurposing.

The research presented above proposes a method for constructing ternary relationships. However, it is acknowledged that these methods may have limitations. For instance, tensor decomposition may not be appropriate for handling large-scale and sparse data sets, and the performance of the event graph model may be restricted by the complex characteristics of nodes when predicting nodes. Therefore, further refinement and optimization may be required to enhance its accuracy and scalability.

Drug repurposing is a cost-effective approach to drug development that could use deep learning models such as graph neural networks, including homograph and heterogeneous graph learning [13]. Homograph graph learning methods concentrate on graphs with nodes and edges of the same type. They leverage graph eigenvalues, eigenvectors [14–18] and spatial features [19–22] for drug repurposing. Additionally, Heterogeneous graphs are used to explore correlations between different types of nodes and edges, providing valuable insights in various fields such as social network analysis [23–25], bioinformatics [26–28], and recommendation systems [29–31].

GNNs have demonstrated effectiveness in tasks such as node classification [32–34], link prediction [35–37], graph classification [38–40], community detection [41–43], and anomaly detection [44–46]. Some GNN models have been developed to meet different graph learning needs [47]. The Graph Convolutional Network (GCN) is commonly used for semi-supervised node classification [48], whereas the Graph Attention Network (GAT) incorporates graph attention mechanisms [49]. The GraphSAGE model uses techniques for graph sampling and aggregation [50], whilst the Graph Isomorphism Network (GIN) focuses on learning at the node and graph level using graph isomorphic operations [51].

GNN has made significant progress in several areas. For instance, the new two-channel hypergraphic convolutional network (HGHDA) model [52] enables the encoding of higher-order relationships between drugs, their constituents and diseases, in order to derive predictions with scoring functions. However, GNN still faces several challenges in drug repositioning. One of the main challenges is effectively linking the relationship among drugs, targets, and diseases so that GNN can learn their interactions. Furthermore, the relationship among drugs, targets, and diseases is complex, requiring GNN to capture and understand multi-level interactions. This involves the interaction between drugs and targets, targets and diseases, and drugs and diseases.

To overcome these shortcomings, a ternary relationship approach to drug repositioning research is presented. By exploring the interactions among drugs, targets and diseases, the underlying mechanisms of drug repurposing can be revealed. Our study creates event nodes that represent the possible relationships among drugs, targets, and diseases. We then establish a map of isomerized event-disease relationships to capture the connections among drugs, targets, and diseases. In addition, a Drug-Target-Disease graph neural network (DTD-GNN) will be constructed to model the relationship among drugs, targets and diseases, and the proposed method will be validated through a link prediction approach. The research aims to provide new insights and methodologies for drug repurposing, contributing to advancements in drug development.

In summary, the main contributions of this work are provided as the following:

- We constructed a dataset based on the relationship among drugs, targets, and diseases, which includes the association information among them. Additionally, we introduced event nodes to represent the ternary relationship among them.
- Based on the associations among drugs, targets, diseases, and events, we constructed a heterogeneous graph structure to represent the intricate relationships between these entities.
- We propose a DTD-GNN model that integrates graph convolutional networks and graph attention networks to learn feature representations and capture association information. This combination enhances the performance of the model, leading to improved results.
- The experimental results demonstrate that the DTD-GNN model outperforms other graph neural network models in various metrics. The model exhibits a superior ability to accurately predict the relationships among drugs, targets, diseases, and events, thereby providing reliable prediction results.

## Methods

In this section, we first introduce the concept of an event node, which represents the ternary relationship among drugs, diseases, and targets. And then explain the modeling process of the event-disease heterogeneous graph. Finally, a new heterogeneous graph is established to represent the relationship between events and diseases. The study further explorers the relationship between these entities through link prediction.

### Construction of event nodes

To enhance our understanding of the relationships among drugs, targets, and diseases, we used an event node to model their interactions. We drew inspiration from Qu et al.’s [12] event graph and developed event nodes to capture and consolidate the relationships among these three entities.

**Fig. 1:**
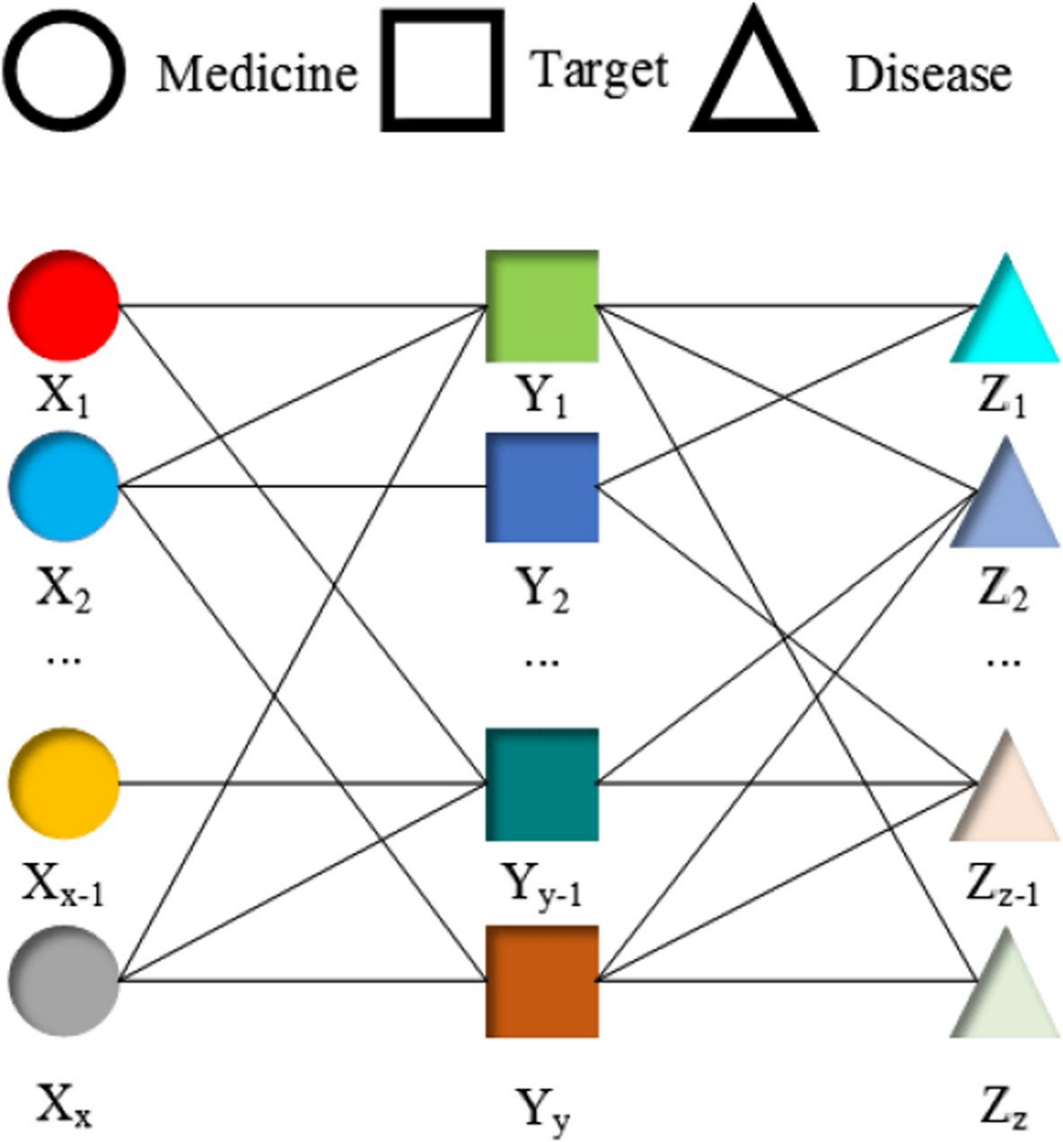
Drug, target, disease interaction Diagram (Paired paradigm)

Given a set of drugs, targets, and diseases, a simple relationship diagram can be constructed to illustrate their interactions, as depicted in Fig. 1. The diagram illustrates the binary relationships between drugs and targets, and targets and diseases. However, this approach fails to capture the interdependent and inseparable nature of drugs, targets, and diseases. To establish a comprehensive and unified understanding of these relationships, the concept of events is introduced.

**Fig. 2:**
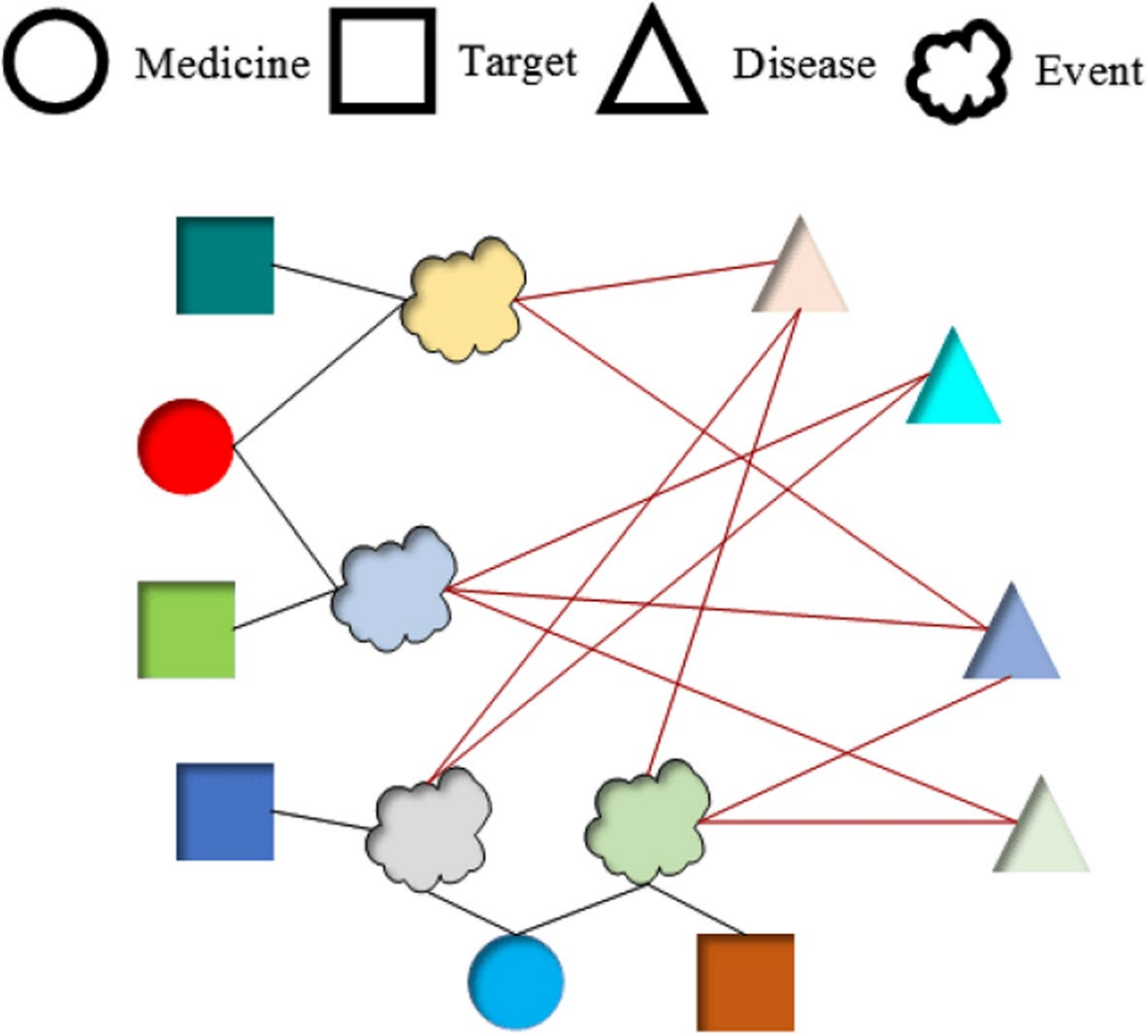
Heterogeneous graph of partial relationship between event and disease. To better represent the inherent combination of drugs and targets within event nodes, we enhance the heterogeneous graph depicting the partial relationship between events and diseases. In addition to the event nodes, we include nodes for drugs and targets in the graph

For all entities in the data (all drugs, targets and diseases are considered as one entity), if there is a relationship among drugs, targets and diseases, that is, if a particular drug $X_i$ and a particular target $Y_i$ are combined so that some corresponding diseases can be treated $Z = \{ Z_1, Z_2, . ., Z_z \}$ (where Z is the collection of disease nodes that can be treated), then the relationship between them can be defined as an event node $Q = <X_i, Y_i, Z>$. For example, in Fig. 2, if the combination of $X_1$ and $Y_1$ can treat $Z = \{Z_1, Z_2, Z_z \}$, then an event $Q = <X_1, Y_1, Z>$ can be constructed.

### Heterogeneous graph construction of event node and disease node

The graph neural network comprises two main types: homograph and heterogeneous graph. In this study, we distinguish between event nodes and disease nodes as distinct types of nodes, enabling us to construct a heterogeneous graph based on their relationships.

In the event node representation, denoted as $Q = <X_i, Y_i, Z>$, and the disease node $Z_i$, we gain an edge between the event node *Q* and the disease node $Z_i$ if $Z_i \in Z$. This edge represents a “link” relationship between the event and the disease. For a clear and intuitive presentation, we present a partial structure of the heterogeneous diagram illustrating the event-disease relationships, as shown in Fig. 2.

### DTD-GNN model construction

Our proposed DTD-GNN model utilises a graph convolutional network to capture the feature relationships between events and diseases, while using a graph attention network to handle the ternary feature relationships. A gate unit is employed to extract relevant features and obtain a new result for feature processing. The model is comprised of the following components:

#### Data feature construction

This section will focus solely on the construction of feature initialization for events, as the association between event and disease has already been elaborated.

To ensure the randomness and diversity of the disease types studied, we employ a random construction method for disease feature construction, as the constructed relationship is an event-disease association. We construct the event feature according to our definition of an event and combine it with the randomly initialized event feature during model training. This approach enables the full utilization of prior knowledge during training, promoting robustness and mitigating overfitting when extracting event features. Our construction method adopts the One-Hot encoding technique, which is explained below:

- First, we create a feature matrix *A* of size $q \times z$ for the identified event and disease types. All elements of this matrix are initially set to 0.
- Next, for each row in the feature matrix *A* (corresponding to each event), we iterate over each column to determine the presence or absence of disease associations. Specifically, if $Z_j \in Q_i$ ( $i \in \{0,1,... ,q\}$, $j \in \{0,1,... ,z\}$), we set the element $A_{ij}$ to 1. Otherwise, if there is no association between the event and disease, $A_{ij}$ remains 0.
- To generate the event feature matrix *A* of size $q \times z$, each row of events is looped through until all events have been traversed. During each iteration, the corresponding row in the feature matrix *A* is updated based on the associations between the events and diseases. Once all events have been iterated through, the event feature matrix *A* is constructed, where each row represents an event and each column represents a disease type. The matrix $A_{q \times z}$ reflects the event-disease associations captured.

#### Encoder design

We employ graph convolutional and graph attention networks to extract features from event-disease and ternary relationships. Gate units [53] optimise the features, and residual connections facilitate feature fusion.

**Fig. 3:**
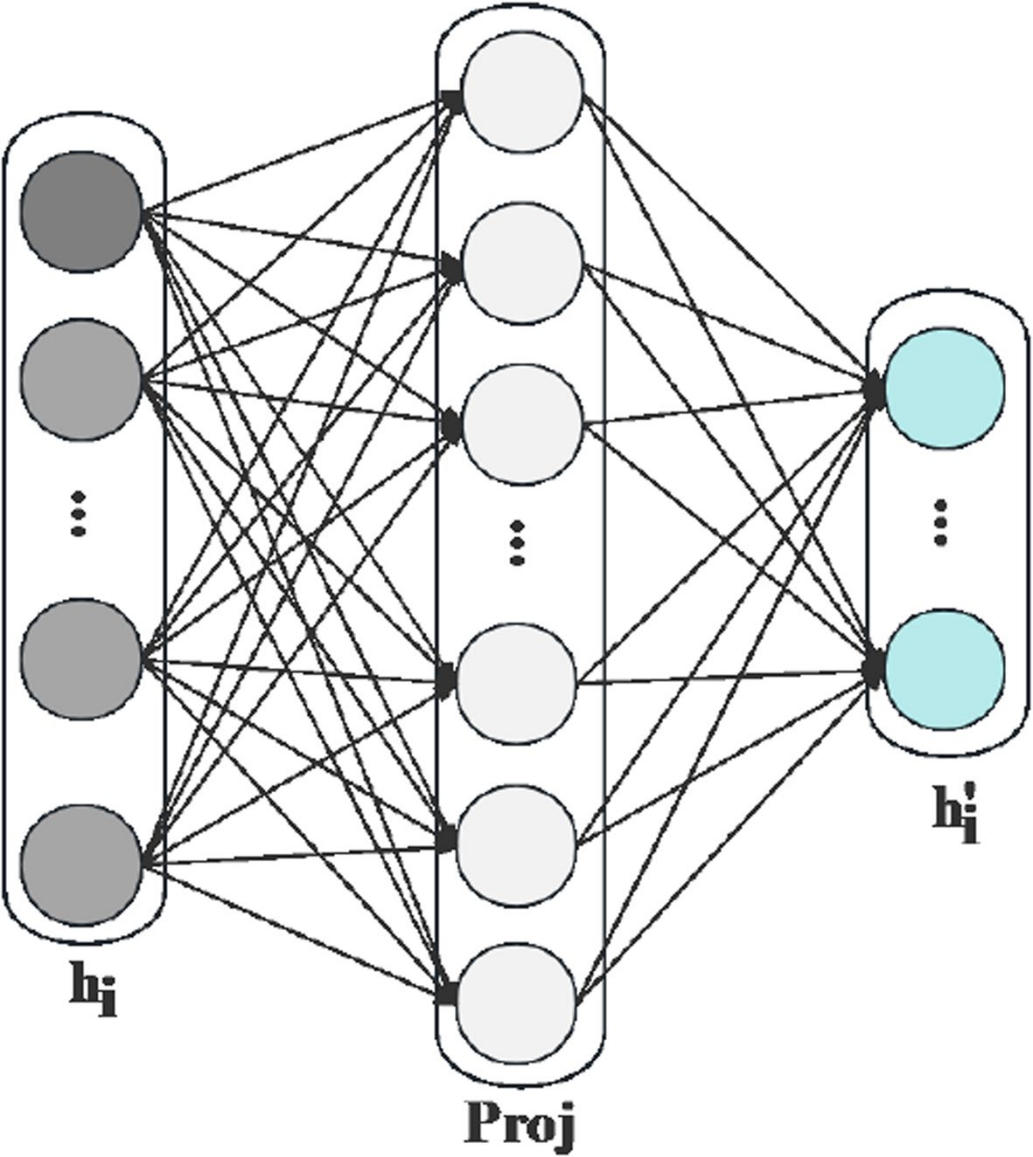
Node feature dimension mapping graph

- Mapping of feature dimensions To ensure that event nodes and disease nodes share the same dimensional features, their node features must be mapped to a common space. For the category of nodes $h_i$, we apply the transformation matrix *M*(*Proj*), as shown in Fig. 3. The mapping formula is as follows:
  1 $$\begin{aligned} h^{\prime }_i = M \cdot h_i \end{aligned}$$
- Convolution and attention of node dimension The graph convolution method is used to process the convolution operation on the nodes, which involves sampling and aggregating nodes. Firstly, a specific number of nodes are randomly sampled from the neighbors of each node *i*, denoted as *N*(*i*). Then, the feature of each node *i*, $h^{\prime }_i$ is aggregated with the features of its neighboring nodes $h^{\prime }_{N(i)}$. The aggregation function can be expressed as follows:
  2 $$\begin{aligned} Z\left( h^{\prime }_i\right) = AGGREGATE\left( \left\{ h^{\prime }_i\right\} \cup \left\{ h_u \, for \, u \, in \, h^{\prime }_{N(i)}\right\} \right) \end{aligned}$$
  Where AGGREGATE is the aggregation function, $h^{\prime }_i$ and $\left\{ h_u \, for \, u \, in \, h^{\prime }_{N(i)}\right\}$ represent the characteristic of node *i* and the feature set of neighbour node *N*(*i*) respectively. Finally, the new representation of node *i*, ${h}^{\prime \prime }_{i}$ is obtained by concatenating the aggregated features $Z\left(h^{\prime }_i\right)$ with the current representation $h^{\prime }_i$ of node *i*. The concatenated features are then mapped through a fully connected layer and activation function. This mapping operation can be expressed as follows:
  3 $$\begin{aligned} {h}^{\prime \prime }_{i} =\sigma \left(W \cdot CONCAT\left({h}^{\prime }_{i},Z\left({h}^{\prime }_{i}\right)\right)\right) \end{aligned}$$
  Where *W* is the weight matrix of the fully connected layer, *CONCAT* represents the join or concatenation operation, and $\sigma$ denotes the activation function. The graph attention mechanism is introduced in the attention operation on the node to extract features using different attention weights. The attention mechanism is defined for each node type *v* to capture the semantic relationships between nodes. It is then used to calculate the attention weight between node *i* and its neighbor node *j*:
  4 $$\begin{aligned} e^{v}_{ij} = \sigma \left(a^{T}\left[ W^{v} h^{v}_{i} || W^{v} h^{v}_{j} \right] \right) \end{aligned}$$
  Where $W^{v}$ is the weight matrix for node type *v*, *a* denotes the parameter vector for the attention mechanism, || and $\sigma$ respectively represent the link operation and the activation function. Then, for each node *i*, the attention weight is utilized to aggregate the neighboring nodes, resulting in a semantic-level representation. This process combines the features of the neighboring nodes based on their attention weights, resulting in a comprehensive representation.
  5 $$\begin{aligned} h^{{v}^{\prime } }_{i} = \sum \limits _{j \in N_{i}^{v} } softmax\left( e^{v}_{ij}\right) *\left(W^{v} h^{v}_{j}\right) \end{aligned}$$
  Where $N^{v}_{i}$ indicates the set of neighbor nodes of type v of node *i*. Finally, for each node *i*, its node-level representation and semantic-level representation can be merged to obtain the final node representation. This fusion process combines the local information from the node-level representation and the semantic information from the semantic-level representation, resulting in a comprehensive and enriched representation that captures both the specific characteristics of the node and its semantic relationships within the graph.
  6 $$\begin{aligned} {h}^{\prime \prime }_{i} = \sigma \left( CONCAT\left( h^{v}_{i}, h^{{v}^{\prime }}_{i} \right) \right) \end{aligned}$$
  Where *v* indicates the type of node *i*, *CONCAT* is a concatenation or merging operation.
- Gate unit The gate unit is a specialised structure used to control information flow and filtering in convolution. These units regulate information and memory flow through activation functions and dot product operations. The introduction of gate units enables the model to capture long-term dependencies more effectively and achieve superior performance in processing feature information. The formula for calculating the gate unit can be expressed as follows:
  7 $$\begin{aligned} h^{\prime }_{i} = h_{i} \cdot \sigma ( W_{i} \cdot h_{i}+ b ) \end{aligned}$$
  Where $h_{i}$ is the final output value, $W_{i}$ and *b* are a learnable weight matrix and a learnable bias vector, $\sigma$ represents the sigmoid activation function.

#### Decoder design

**Fig. 4:**
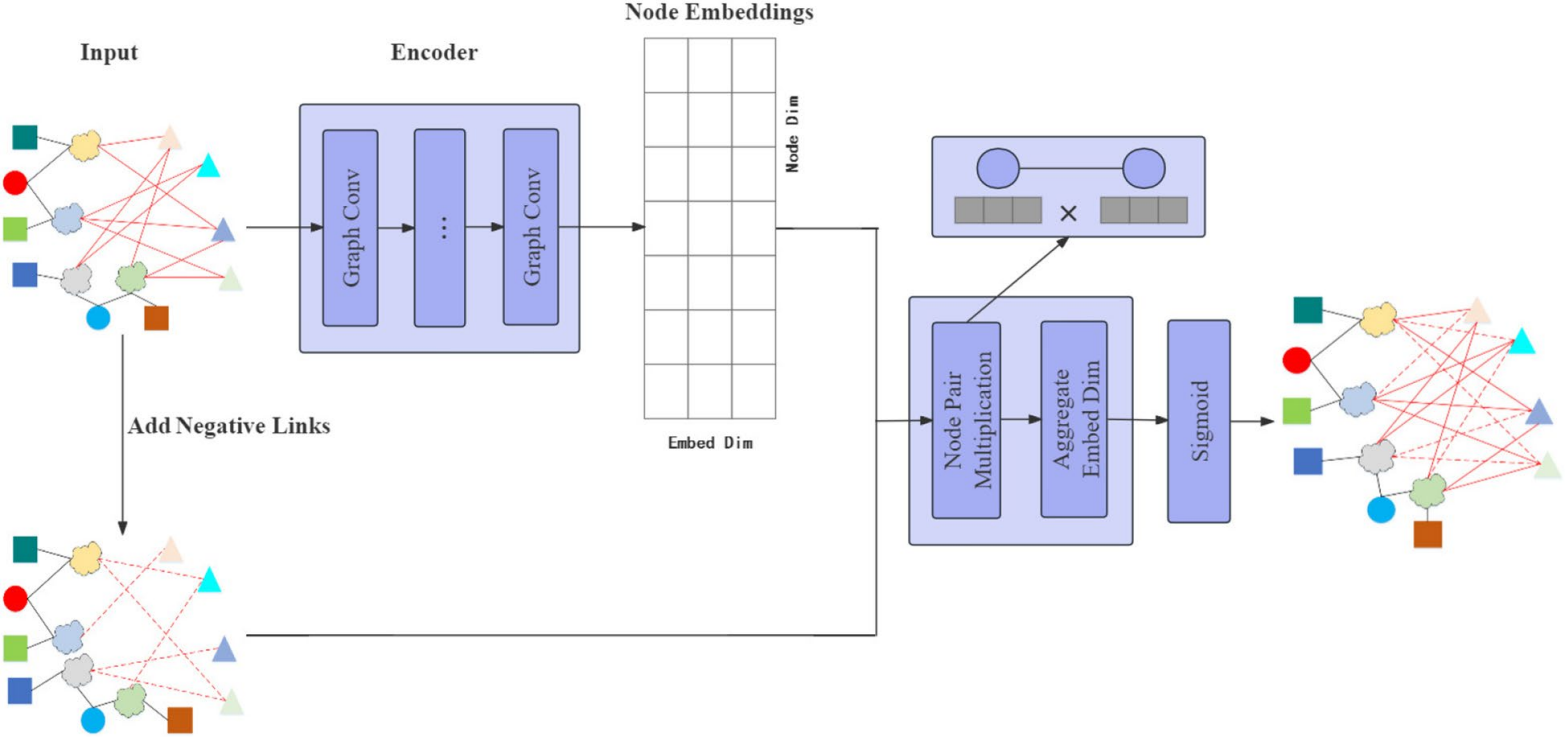
Link prediction flowchart, where Node Pair Multiplication represents the dot product of the compute node embeddings and Aggregate Embed Dim represents the value of aggregating the entire embeddings dimension

When studying the relationship between events and diseases, link prediction is used to assess the potential association between the two. Link prediction is a more intricate task than node classification as it involves using node embeddings to predict edges in a graph. The process of link prediction is illustrated in Fig. 4.

- First, The node embeddings are generated by the encoder through the application of *L* convolutional layers to process the input graph.
- Then, to enhance the link prediction task, negative links are randomly added to the original graph. Negative links are crucial in predicting associations in graph neural networks. They represent negative correlations between events and diseases, reflecting exclusion or inhibition mechanisms. Modeling these negative links helps the DTD-GNN model learn detailed relational information, improving link prediction accuracy and robustness. Additionally, negative links aid in identifying anomalous or noisy relationships, enabling the model to differentiate genuine negative correlations from biased spurious relationships. This modification transforms the task into a binary classification problem. The model must distinguish between the positive links from the original edges and the negative links from the newly added edges.
- Finally, the decoder uses node embeddings to predict links, performing binary classification for all edges, including negative ones. It calculates the dot product between the node embeddings of a pair of nodes on each side and aggregates the values across the entire embedded dimension. This generates a probability value representing the likelihood of the edge’s existence for each edge.

#### Loss function and optimizer selection

The BCEWithLogitsLoss loss function is appropriate for binary classification problems, which is applicable to the link prediction task in our drug repurposing project. This task involves determining whether a link exists between a given event and a disease. The BCEWithLogitsLoss function effectively handles this binary classification scenario. In drug repurposing link prediction, positive and negative samples are often imbalanced, with a significantly larger number of negative samples compared to positive samples. To balance the influence of positive and negative samples and enable better handling of unbalanced data, BCEWithLogitsLoss can be weighted for different sample categories.

The Adam optimizer is suitable for the drug repurposing link prediction task because of its adaptive learning rate adjustment based on the gradient range and parameter variations. In this task, different event-disease relationships may exhibit diverse gradient properties, and Adam can automatically adapt the learning rate to optimize performance in various scenarios. Furthermore, the Adam optimizer exhibits rapid convergence in the early stages of training, often achieving superior results in fewer iterations. Moreover, the Adam optimizer performs well when handling large-scale data and parameters, effectively optimizing the model.

**Fig. 5:**
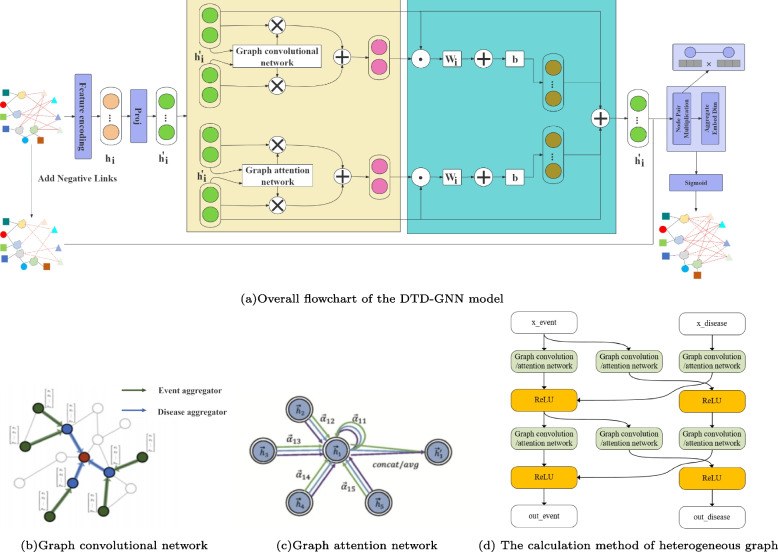
DTD-GNN model flow diagram, where (**b**) and (**c**) correspond to the network structure in the diagram, and (**d**) corresponds to the calculation of features between nodes in the yellow section

We have successfully developed our DTD-GNN model based on the information provided. The model’s complete process is illustrated in Fig. 5.

## Experiments

### Data preparation

We collect paired records from the publicly available BioSNAP dataset [54], which contains information on drug-target, drug-disease and target-disease relationships. We extract a total of 15,140 drug-target, 4,66,658 drug-disease and 15,509,620 target-disease pairs from the BioSNAP dataset. These pairs represent a one-to-one correspondence between drugs and targets, drugs and diseases, and targets and diseases. By merging these pairs based on their relationships, we create a ternary relationship known as the drug-disease-target relationship, which forms the event node. The detailed statistics of the paired datasets are shown in Table 1, and the node statistics and event-disease relationships are shown in Table 2.

**Table 1:** The number of paired relationships between drugs, targets and diseases

Description	Drug-Target	Drug-Disease	Target-Disease
Number	15140	466658	15509620

**Table 2:** The number of nodes and event-disease relationships

Description	Drug	Target	Disease	Event	Event - Disease
Number	1318	1360	3111	7357	2170264

A heterogeneous graph is constructed based on the correlations between events and diseases. The edge set of the dataset is divided into three subsets for training, validation, and testing purposes. The training set is allocated $60\%$ of the edges, the validation set is allocated $10\%$, and the remaining $30\%$ is allocated to the test set. The results are then experimentally validated using these subsets.

### Experimental verification

The experiment consists of four main components. Firstly, the optimal parameter settings for the DTD-GNN model are determined through parameter adjustment and training, using the BioSNAP dataset. Secondly, an ablation experiment is conducted on various aspects of the DTD-GNN model to identify the optimal structure. Additionally, we validated our approach by treating the relationship between events and diseases as link prediction. We compared our model’s link prediction performance with other models’ node classification performance using the AUC indicator. Finally, we selected heterogeneous graph neural network models commonly used for link prediction to demonstrate the advantages of our model. The next section will explain these indicators.

- Accuracy is a metric that measures the percentage of correctly predicted results out of the total sample.
  8 $$\begin{aligned} Accuracy = \frac{TP+TN}{TP+TN+FP+FN} \end{aligned}$$
  Where TP stands for true positive, indicating that the prediction is true and aligns with the actual positive class. FP refers to false positive, signifying that the prediction is true, but it contradicts the actual negative class. FN represents false negative, indicating that the prediction is false, despite the actual class being positive. TN means true negative, denoting that the prediction is false, and it aligns with the actual negative class.
- The accuracy rate, also known as the precision rate, represents the probability that a sample predicted as positive is indeed positive.
  9 $$\begin{aligned} Precision = \frac{TP}{TP+FP} \end{aligned}$$
- The recall rate, and known as the sensitivity or true positive rate, represents the probability of a positive sample being correctly predicted as positive.
  10 $$\begin{aligned} Recall = \frac{TP}{TP+FN} \end{aligned}$$
- The F1 value is a metric that considers both the precision rate and recall rate simultaneously, aiming to achieve a balanced performance.
  11 $$\begin{aligned} F1 = \frac{2*Precision*Recall}{Precision+Recall} \end{aligned}$$
- The AUC (Area Under the Curve) index is calculated by measuring the area under the Receiver Operating Characteristic (ROC) curve, which is commonly used for comparing the performance of different models. The AUC value serves as an indicator of model performance, with larger values indicating better performance. Typically, the AUC value ranges between 0.5 and 1. The ROC curve is a graphical tool used to evaluate the performance of binary classification models. It plots the true positive rate (sensitivity) on the vertical axis against the false positive rate (1 - specificity) on the horizontal axis. The True Positive Rate (Sensitivity) is the proportion of correctly predicted positive instances out of all actual positive instances.
  12 $$\begin{aligned} True\ Positive\ Rate = \frac{FP}{FP+TN} \end{aligned}$$
  The False Positive Rate (1 - Specificity) is the proportion of incorrectly predicted negative instances out of all actual negative instances.
  13 $$\begin{aligned} False\ Positive\ Rate = \frac{TP}{TP+FN} \end{aligned}$$
- The AUPR metric is frequently used to assess the performance of binary classification models, especially for tasks that involve imbalanced datasets or a focus on positive samples. It measures the balance between precision and recall demonstrated by the model across different thresholds. Unlike the ROC curve and AUC, which evaluate model performance based on the true class rate and false positive class rate, the AUPR assesses the relationship between accuracy rate and recall rate. The AUPR value ranges from 0 to 1, with higher values indicating superior model performance.

To evaluate the indicators mentioned above, we conduct experiments and obtain results. The details of our experimental environment are presented in Table 3.

**Table 3:** Experimental environment configuration

Description	Configuration	
Hardware environment	GPU	RTX 3090(24GB) * 1
	CPU	15 vCPU Intel(R) Xeon(R) Platinum 8350C CPU @ 2.60GHz
	Memory	56GB
	Hard disk	System disk 30 GB
		Data disk 50GB SSD
Software environment	Operating system	ubuntu20.04
	Python	3.8
	Cuda	11.8
	Core Libraries	torch-geometric 2.4.0
		PyTorch 2.0.0
		Numpy 1.24.2
		Scikit-learn 1.3.2

## Results and analysis

### DTD-GNN model parameters

We perform tuning and iterative training on our model using the experimental environment and the BioSNAP dataset. This process entails optimizing seven crucial parameters: learning rate, loss function, optimizer, batch size, dimension number of node embedding, dropout rate, and weight decay. After extensive experimentation and continuous adjustments, we arrive at the final set of parameters for the trained DTD-GNN model. These parameters are presented in Table 4 below.

**Table 4:** Parameters of the DTD-GNN model

Parameters of the DTD-GNN model	Parameter value
Learning Rate (lr)	1e-3(0.001)
Loss function	BCEWithLogitsLoss
Optimizer	Adam
Batch size	3840
embedding dimension	64
Dropout rate	0.6
Weight decay	1e-5(0.00005)

### Ablation experiment

We conduct an ablation experiment on the core components of our DTD-GNN model to evaluate their impact on overall performance. The experiment focused on several factors, including the number of convolution layers (ranging from 1 to 3 layers), the separation and merging of the graph convolution layer and graph attention layer, and different decoding methods (such as feature product fusion, unilinear feature fusion, and bilinear feature fusion).

The results of the experiments are presented in Table 5, with values rounded to five decimal places. The best results are highlighted in bold. For each ablation experiment, the default setting indicates that the structure of the other model components remained unchanged while only one component’s structure was modified.

**Table 5:** Ablation experiment results

Component of model	Class of components	Result					
		AUC	Accuracy	F1	Precision	Recall	AUPR
Convolution layer	One-layer convolution	0.98620	0.91782	0.91233	0.97765	0.85519	0.98316
	Three-layer convolution	0.98655	**0.91821**	0.91282	0.97723	**0.85637**	0.98342
Diagram of the network structure	Separate graph convolutional network	0.97075	0.89511	0.88949	0.93987	0.84423	0.96602
	Separate graph attention network	0.96569	0.86249	0.84808	0.94736	0.76763	0.95802
Decoder	Single-linear feature fusion	0.96307	0.88181	0.87452	0.93195	0.82376	0.96117
	Double line feature fusion	0.98551	0.91636	0.91075	0.97629	0.85346	0.98180
DTD-GNN: Convolution layer: Two convolution layers Graph network structure: graph convolutional network + graph attention network + gate unit Decoder: feature product fusion		**0.98687**	0.91212	**0.90540**	**0.98039**	0.84106	**0.98437**

Table 5 shows that the performance of the DTD-GNN model is significantly impacted by different component structures. The number of convolution layers has minimal effect on the model’s performance, with most indicators showing insignificant changes. However, increasing the number of convolutional layers to three reveals interesting phenomena. Specifically, the addition of a convolutional layer results in a decrease in Precision and an increase in Recall. Precision represents the proportion of predicted positive samples that are actually positive, while Recall represents the proportion of correctly predicted true positive samples. This suggests that the DTD-GNN model focuses more on quantity rather than quality when predicting positive examples. Based on this analysis, we select two convolutional layers as the optimal component structure. Our goal is to predict as many correct samples as possible while maintaining a high level of accuracy.

The experimental comparison was made between two different graph network structures: graph convolution network and graph attention network. The results indicate that the performance of the six indicators has significantly decreased when compared to the DTD-GNN model. This is due to the limitations of using a convolution network alone to process drug-target-disease data. The convolution network can only capture local neighborhood information in graph data, potentially ignoring the relationship between distant nodes. This limitation can lead to the convolution network failing to make full use of global information in complex drug-target-disease relationship networks, ultimately affecting prediction performance. Additionally, using graph attention network alone has limitations in processing drug-target-disease data. While it can adjust the weight between nodes as needed, its computational complexity is high, particularly when dealing with large-scale graph data. This can result in low training and reasoning efficiency, which restricts its practical application. Additionally, it is challenging to model long-distance relationships. While attention mechanisms can capture global information to some extent, they may not transmit enough information when dealing with nodes that are far apart. This is because the attention weight may be attenuated or diluted during the propagation process, leading to limited information exchange between distant nodes.

In our experiments, we evaluated two additional decoders: the singlinear feature fusion decoder and the bilinear feature fusion decoder. The singlinear feature fusion decoder showed significant performance degradation compared to the DTD-GNN model across six metrics, indicating its ineffectiveness for drug repurposing tasks. However, the bilinear feature fusion decoder exhibits slightly lower performance in certain metrics compared to the DTD-GNN model’s product feature fusion decoder. Nevertheless, the overall difference is not substantial, indicating that the bilinear feature fusion decoder has potential for drug repurposing tasks and may offer advantages in specific scenarios. Based on the experimental findings, however, the product feature fusion decoder remains the preferred choice. It is demonstrated that this decoder outperforms others, effectively utilizes feature information, and is suitable for predicting drug repurposing tasks.

During the investigation of the gate unit, we conducted experiments to assess its significance by removing it from the model. However, the experimental results showed a consistent downward trend across six key metrics after the removal of the gate unit. The gating unit controls the flow of information through the learned weights, allowing for more accurate capture of the key information in the graph data. By introducing the gating unit, the model’s perception of global information can be improved, enhancing its flexibility and scalability. The gating unit can adjust the information transmission intensity between nodes, optimizing the model’s performance for the task. Removing the gating unit reduces the model’s expressiveness and generalization capabilities, leading to a decline in the accuracy of predicted results.

### Comparison experiment

 

**Fig. 6:**
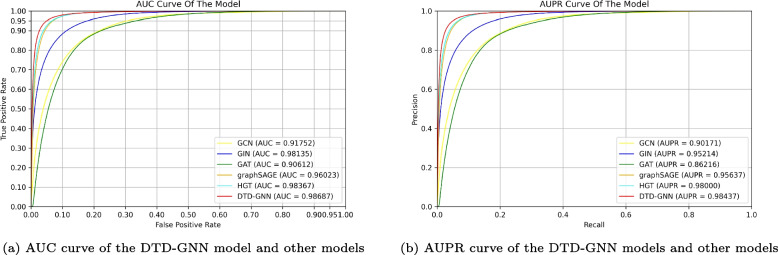
AUC and AUPR curves of the model

- Comparison experiment between node classification and link prediction task In our study, we treat the event-disease relationship as a link prediction task. Graph neural networks are commonly used for node classification and link prediction in drug relocation tasks. To demonstrate the effectiveness of this approach, we conduct a comparative experiment using eight widely graph neural network models. We ensure a fair comparison by selecting these models: GCN [48], GraphSAGE [50], GIN [51], HIN2Vec [55], HGT [56], Event2Vec [57], HGNN [58], and EGNN [12]. The test results of these models are compared with the link predictions of our DTD-GNN model using the AUC metric. The experimental results, presented in Table 6, display the results rounded to three decimal places, with the best-performing model highlighted in bold. The data presented in Table 6 indicates that using graph neural network models for node classification tasks yields inferior results compared to link prediction tasks when studying the relationship between events and diseases. The aim of the node classification task is to categorise nodes within a graph. However, accurately distinguishing between categories can be difficult due to limited information on node connections and features, particularly for complex heterogeneous graphs. Events are entities with a structured format that involve ternary relationships. Therefore, the relationship between event nodes and disease nodes can be intricate, and the expression of node features may be incomplete or contain noise. Link prediction leverages existing connection patterns to predict connections between unlinked nodes, allowing for the inference of potential relationships based on the graph’s connectivity information. Graph neural network models are ideal for link prediction tasks as they produce superior results in studying the relationship between events and diseases.
- Comparison experiment of models in link prediction tasks To assess the effectiveness of our DTD-GNN model in predicting links, we compared it with other commonly graph neural network models. For this analysis, we chose five models: GCN [48], GraphSAGE [50], GAT [26], GIN [51], and HGT [56]. Additionally, we used publicly available code to train, validate, and test the model on the dataset. The experimental results are presented in Table 7 with five decimal places. The best-performing results are highlighted in bold. Additionally, the AUC and AUPR curves of the models are visualised in Fig. 6, providing insights into the models’ performance across different thresholds. The experimental results presented in Table 7 demonstrate that our DTD-GNN model outperforms other models in predicting event-disease relationships. It excels in evaluation metrics such as AUC, F1-score, and Precision. Additionally, AUC and AUPR graphs highlight its superior predictive ability compared to other models. The DTD-GNN model efficiently integrates information from drugs, targets, and diseases, capturing complex relationships and enabling accurate predictions in drug repositioning. The evaluation of the DTD-GNN model shows a slight decrease in accuracy and recall compared to the GraphSAGE model across the six metrics. However, this difference can be interpreted in the context of the specific objectives of the DTD-GNN model. The primary focus of the DTD-GNN model is to study the relationship between events and diseases, and predict this relationship by constructing a heterogeneous graph structure involving drugs, targets, and diseases. The primary aim of this task is to ensure high precision and confidence in predicting event-disease relationships. To achieve this, emphasis should be placed on the quality of the predicted positive samples. Precision is a measure of the accuracy of our predicted positive samples, which reflects the overall quality of our predictions. Recall measures the proportion of correctly identified positive samples out of all actual positive samples, prioritising quantity over quality. However, in the specific context of studying event-disease relationships, it is crucial to ensure the precision of high-quality associations rather than identifying a larger number of low-quality associations. Therefore, although the DTD-GNN model may exhibit relatively lower values in terms of accuracy and recall, it excels in precision and provides high-confidence predictions when studying the relationship between events and diseases. Overall, it outperforms the GraphSAGE model in this task, particularly in terms of predicting the quality and confidence of positive samples. The DTD-GNN model’s optimisation towards task-specific objectives enhances its value and usefulness in addressing the drug repositioning problem. In drug repositioning tasks, it is common to encounter class imbalance issues, where there is an unequal distribution of positive and negative samples. In such scenarios, relying solely on accuracy as an evaluation metric is insufficient to provide a comprehensive assessment of model performance. This limitation arises from the potential bias of the model towards predicting a larger number of samples as the majority class, which can lead to biased predictions, particularly for negative samples. Therefore, the accuracy metric may be compromised and may not accurately reflect the overall effectiveness of the model. The DTD-GNN model is proficient in acquiring unique representations of diseases and events. It efficiently employs association information to capture complex relationships, resulting in comprehensive feature representations and improved performance. The model gains a deeper understanding of the intrinsic characteristics of these entities by utilizing feature embedding techniques, enabling accurate capture of their associations and enhancing predictive performance. The evaluation metrics, namely AUC, F1, and Precision, demonstrate the beneficial effects of feature learning.

**Table 6:** The experimental results comparing the node classification task and the link prediction task

Task type	Graph neural network model	AUC
Node classification	GCN [48]	0.914
	GraphSAGE [50]	0.923
	GIN [51]	0.916
	HIN2Vec [55]	0.689
	HGT [56]	0.882
	Event2Vec [57]	0.663
	HGNN [58]	0.905
	EGNN [12]	0.932
Link prediction	DTD-GNN	**0.987**

**Table 7:** The comparative experimental results of the models in the link prediction task

Graph neural network model	Result					
	AUC	Accuracy	F1	Precision	Recall	AUPR
GCN [48]	0.91752	0.80270	0.77701	0.89331	0.68750	0.90171
GraphSAGE [50]	0.98135	**0.93491**	0.90045	0.91840	**0.88319**	0.95214
GAT [26]	0.90612	0.79392	0.76758	0.88005	0.68060	0.86216
GIN [51]	0.96023	0.85886	0.84427	0.94163	0.76515	0.95637
HGT [56]	0.98367	0.92087	0.91696	0.96463	0.87377	0.98000
DTD-GNN [12]	**0.98687**	0.91212	**0.90540**	**0.98039**	0.84106	**0.98437**

### Visual presentation of the DTD-GNN model

**Fig. 7:**
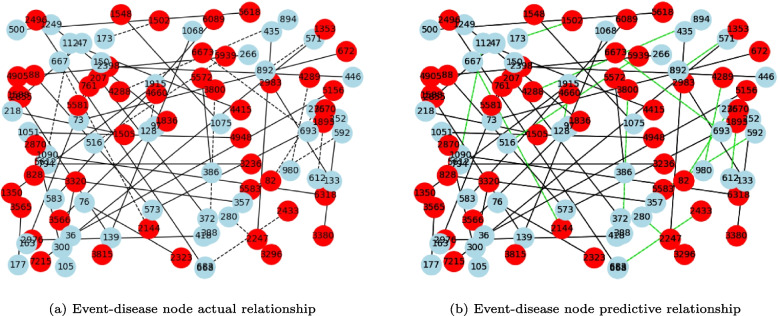
The figure presents a diagram showcasing the relationship between event and disease nodes. Disease nodes are denoted by the color blue, while event nodes are represented by the color red. In (**a**) graph, it displays the initial link between events and diseases, with dotted lines indicating the entity relationships requiring prediction. The (**b**) graph illustrates the predictions made by our DTD-GNN model for the event-disease link. The solid green lines represent the discernment results of the DTD-GNN model regarding the predicted entity relationships

We have conducted visualizations of the predictions made by the DTD-GNN model. Considering the large number of nodes and links between events and diseases, we have selected 100 link relationships for visualization purposes. This enables direct observation and examination of the connections between events and diseases, as depicted in Fig. 7.

The analysis of Fig. 7 shows a clear correspondence between the prediction effect diagram of our DTD-GNN model and the actual relationship diagram. The visualization provides an intuitive understanding of the mutual relationships between event nodes (depicted as red nodes) and their corresponding disease nodes (depicted as blue nodes). These observations provide additional evidence for the effectiveness of our DTD-GNN model in accurately capturing and representing the connections between events and diseases. The relationship diagram uses solid lines to represent the relationship information used for model training. Dotted lines illustrate the relationship information between nodes that require prediction during model testing. The green color in the prediction effect diagram signifies the successful predictions made by the DTD-GNN model regarding the test edges. Based on the diagrams, it can be concluded that the DTD-GNN model accurately predicts the edges of the test data with a high degree of certainty.

## Case study

Based on the outstanding performance of our framework, we have selected event No.2637 (drug DB00543, namely Amoxapine, is a tricyclic antidepressant used in the treatment of neurotic or reactive depressive disorders and endogenous or psychotic depression, target P50406) for analysis to investigate its potential relationship with various diseases. The model was pre-trained on BioSNAP data to predict the existence of a relationship between current events and diseases.

The model’s prediction for event No.2637 indicates a connection between the event and 100 out of 3111 diseases. This suggests a relationship between the event and these 100 diseases. The predicted disease results associated with this event closely align with the actual correlation, indicating that the combination of the DB00543 drug and P50406 target in this event has a positive impact on the treatment of these 100 diseases. Table 8 displays the 100 diseases associated with this event.

**Table 8:** Diseases related to the event No.2637

Event	Diseases		
No.2637 (drug DB00543, target P50406)	MESH:D013610	MESH:D019966	MESH:D001714
	MESH:D000740	MESH:D002658	MESH:D000230
	MESH:D007172	MESH:D008569	MESH:D012640
	MESH:D012559	MESH:D019970	MESH:D006930
	MESH:D004409	MESH:D001289	MESH:D006333
	MESH:D007018	MESH:D011470	MESH:D014029
	MESH:D009069	MESH:D000568	MESH:D010698
	MESH:D007022	MESH:D008180	MESH:D000236
	MESH:D005687	MESH:D007249	MESH:D003865
	MESH:D013375	MESH:D007174	MESH:D020018
	MESH:D017109	MESH:D005334	MESH:D008325
	MESH:D019969	MESH:D009459	MESH:D002375
	MESH:D001321	MESH:D004421	MESH:D007006
	MESH:D009410	MESH:D015430	MESH:D006177
	MESH:D006973	MESH:D011471	MESH:D001480
	MESH:D010911	MESH:D012735	MESH:D006966
	MESH:D012516	MESH:D011249	MESH:D006948
	MESH:D001930	MESH:D064420	MESH:D058186
	MESH:D007238	MESH:D015674	MESH:D016171
	MESH:D010300	MESH:D001008	MESH:D020820
	MESH:D005327	MESH:D001049	MESH:D011644
	MESH:D020734	MESH:D001943	MESH:D013226
	MESH:D010146	MESH:D009127	MESH:D015175
	MESH:D003866	MESH:D001919	MESH:D003072
	MESH:D007247	MESH:D006940	MESH:D002819
	MESH:D012206	MESH:D001169	MESH:D004487
	MESH:D004342	MESH:D062787	MESH:D001927
	MESH:D013617	MESH:D014103	MESH:D011537
	MESH:D000647	MESH:D056486	MESH:D006967
	MESH:D012798	MESH:D006212	MESH:D020233
	MESH:D020078	MESH:D003875	MESH:D009290
	MESH:D009437	MESH:D016055	MESH:D005119
	MESH:D020326	MESH:D016535	MESH:D001282
	OMESH:D006556		

Furthermore, the predictions indicate that a combination of drug DB00543 and target P50406 can be used to treat two common diseases.

- Hypertension(MESH:D006973): Hypertension is a prevalent cardiovascular disorder, the etiology of which is multifactorial. Prolonged unmanaged hypertension increases the risk of cardiovascular complications such as heart disease and stroke. Studies have demonstrated that the drug DB00543 effectively reduces blood pressure by targeting the protein P50406, and is a commonly utilized pharmaceutical for the treatment of hypertension.
- Diabetes(MESH:D003920): Diabetes mellitus is a metabolic disease caused by abnormalities in insulin secretion or utilisation. Drug DB00543 has been shown to enhance insulin sensitivity and improve glucose metabolism by acting on target P50406, and thus plays an important role in the treatment of diabetes mellitus.

## Conclusion

In the paper, we introduce the use of event nodes to establish a ternary relationship among drugs, targets, and diseases. The effectiveness of the proposed event-disease heterogeneity map is evaluated on the BioSNAP dataset. Additionally, a new DTD-GNN model is introduced, which combines graph convolution network and graph attention network to accurately represent the complex relationship between drugs, targets, and diseases through feature representation learning on heterogeneous graphs.

The DTD-GNN model demonstrated impressive performance across various classification metrics. These results validate the effectiveness and superiority of our model in exploring the relationships between drugs, targets, and diseases. The study has significant reference value for understanding the mechanism of drug action on diseases, drug repositioning, disease treatment, drug development, and treatment strategies in related fields. Additionally, the research contributes to the advancement and implementation of optimising drug design, drug screening, and drug repositioning. This provides guidance for improving drug effectiveness and reducing side effects.

## Data Availability

All data generated or analysed during this study are included in this published article. All necessary data sets are available and publicly accessible on the Stanford Biomedical Network Dataset Collection (http://snap.stanford.edu/biodata/).
